# Perimesencephalic non-aneurysmal subarachnoid hemorrhage: is there a need for repeat digital subtraction angiography?

**DOI:** 10.1007/s00234-025-03779-8

**Published:** 2025-09-26

**Authors:** Rosa Schubert, Maharani Budi Santoso, Yan Li, Cornelius Deuschl, Ramazan Jabbarli, Ulrich Sure, Michael Forsting, Hanna Styczen

**Affiliations:** 1 Institute for Diagnostic and Interventional Radiology and Neuroradiology, University of Duisburg-Essen, Essen, Germany; 2 Department of Neurosurgery and Spine Surgery, University of Duisburg-Essen, Essen, Germany

**Keywords:** Perimesencephalic subarachnoid hemorrhage, Non-aneurysmal subarachnoid hemorrhage, Digital subtraction angiography, Repeat angiograph

## Abstract

**Purpose:**

To assess the diagnostic yield and clinical relevance of repeated digital subtraction angiography (DSA) in patients with spontaneous perimesencephalic non-aneurysmal subarachnoid hemorrhage (PMSAH) and to evaluate DSA-related complication rates.

**Methods:**

Retrospective analysis of 82 patients with PMSAH confirmed by non-contrast CT between March 2002 and February 2025. All patients underwent initial computed tomography angiography (CTA) and first DSA on average within 24 h. If no bleeding source was identified, a second DSA was performed after 10–14 days. Clinical data, radiological findings, and complications were evaluated.

**Results:**

The initial DSA showed no vascular abnormality in 76/82 patients (92.7%). A second DSA was performed in 60/76 cases (78.9%) and identified two small basilar artery aneurysms (3.3%) that were not visible on the initial CTA or DSA but were detectable on repeat 3D rotational angiography (3DRA). Procedure-related complications occurred in three DSAs (2%), including a cerebellar infarct, supratentorial embolism, and local puncture site complication. Most patients had a benign clinical course, and only one in-hospital death was documented.

**Conclusion:**

The diagnostic benefit of repeat DSA in typical PMSAH is low, with no clear therapeutic impact and a relevant risk of complications. Given the benign course of PMSAH and the availability of high-resolution non-invasive imaging such as dual-source, photon-counting CTA, or vessel wall MRI, repeat DSA should be reserved for selected cases with atypical features or clinical deterioration.

## Background

Approximately 85% of spontaneous subarachnoid hemorrhages (SAH) occur due to the rupture of an intracranial aneurysm [1]. Despite comprehensive imaging examinations, the exact origin of bleeding remains unknown in approximately 15% of cases, termed non-aneurysmal subarachnoid hemorrhages [2].

Regarding computed tomography (CT) findings, non-aneurysmal SAH is categorized into two primary types: perimesencephalic SAH (PMSAH) and non-perimesencephalic SAH (NPMSAH). PMSAH is characterized in non-contrast CT by blood distribution in the prepontine cistern extending to the perimesencephalic area, typically without intraventricular or parenchymal hemorrhage [3]. Subsequently, a computed tomography angiography (CTA) is performed, followed by a digital subtraction angiography (DSA), which is the gold standard for identifying aneurysms in patients with non-traumatic SAH [4].

A standardized diagnostic approach for PMSAH remains elusive, with significant variability in practice across different centers. While some institutions strictly follow guidelines, others adopt more individualized protocols based on local expertise and available resources. The need for additional testing, particularly repeat DSA, remains widely debated, and existing guidelines do not provide uniform recommendations. The European guidelines advise against a second DSA if the initial examination is negative [5], whereas the American Heart Association guidelines remain inconclusive, advocating to proceed cautiously when deciding to repeat imaging [4].

Considering the generally benign course and favorable prognosis of PMSAHs, the necessity of initial and repeated DSA remains a topic of discussion [6–10].

Neuroradiologists and neurosurgeons also continue to debate whether to omit DSA for PMSAHs and opt for CTA instead [11]. The identification of an aneurysm in PMSAHs could have significant therapeutic implications.

Therefore, balancing the potential benefits of detecting a hidden aneurysm against the risks of invasive imaging remains a key challenge, leading to differences in practice between institutions [12–14]. At our institution, a second DSA is performed 10 to 14 days later if the initial CTA and DSA results are negative. Our study evaluates whether this follow-up DSA identifies findings requiring intervention while also assessing the risk of DSA-related complications.

## Methods

We conducted a retrospective study of patients with PMSAH identified on initial non-contrast CT between March 2002 and February 2025.

Patients with non-traumatic SAH visible on CT were included, whereas those with positive cerebrospinal fluid findings but no CT evidence of bleeding were excluded.

**Fig. 1:**
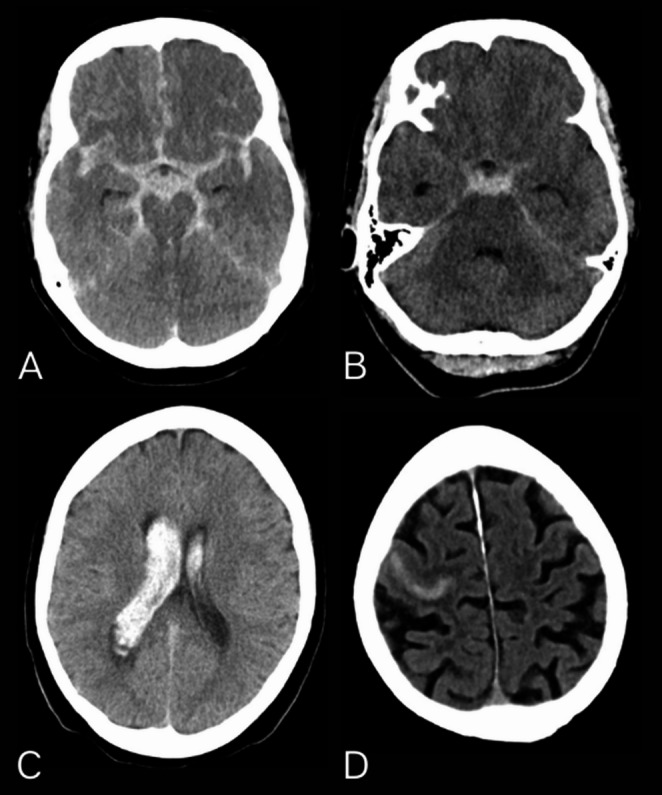
Non-contrast cranial CT examples of subarachnoid hemorrhage. **A**. Diffuse SAH: Hemorrhage is observed within the interhemispheric fissure, extending into both lateral Sylvian fissures **B.** Perimesencephalic SAH: Blood is concentrated in the interpeduncular cistern and extends into the basal Sylvian fissure **C.** Isolated intraventricular SAH: Hemorrhage is confined to both lateral ventricles, with no blood present in the subarachnoid space or other intracranial regions **D.** Sulcal SAH: Blood is localized to the right precentral sulcus, demonstrating a localized sulcal hemorrhage

Patients were categorized based on their initial CT bleeding pattern into four groups: (1) PMSAH, (2) diffuse SAH, (3) sulcal SAH, and (4) intraventricular bleeding (IVB). Examples of the bleeding patterns are shown in Fig. 1.

A perimesencephalic hemorrhage was defined according to van Gijn and Rinkel et al. [2]. The bleeding is typically located anteriorly to the midbrain with or without spreading to the anterior part of the ambient cistern. It may extend towards the medial Sylvian fissures or the anterior section of the interhemispheric fissure but does not spread to the lateral or entire interhemispheric fissure. While there might be some blood sedimentation in the ventricular system, such as sediment in the occipital horns of the lateral ventricles, the presence of a frank intraventricular hemorrhage or extension into brain tissue is not characteristic of this type of SAH.

According to our hospital protocol, all patients with non-traumatic SAH detected on CT underwent CTA. Regardless of CTA findings, a DSA was performed within 24 h to rule out intracranial sources of the SAH. If an aneurysm was detected in the DSA, immediate treatment was initiated. If the initial DSA was negative, a follow-up DSA was performed after 10 to 14 days, alongside cranial and spinal MRI. For patients with an atypical blood distribution, a third DSA was conducted at a later stage.

Senior physicians specializing in interventional neuroradiology reviewed all non-contrast CT, CTA, and DSA images to assess the presence and characteristics of SAH. Furthermore, patient cases and subsequent procedures were discussed in an interdisciplinary conference involving neuroradiologists and neurosurgeons. A medical doctoral candidate in neuroradiology identified PMSAH patients within the non-traumatic SAH group and consulted a senior interventional neuroradiologist in cases of uncertainty.

Patient characteristics, length of stay, and in-house mortality, along with clinical scores like the Glasgow Coma Scale (GCS), World Federation of Neurosurgical Societies (WFNS) grading, Hunt and Hess, and modified Fisher scale, were documented to assess the initial neurological or radiological status of PMSAH patients. Additionally, SAH-related clinical events (hydrocephalus, vasospasms, ventriculoperitoneal (VP) shunt, ventriculostomy, spinal drainage), patient risk factors (diabetes, obesity, hypertension, alcohol/drug use, anticoagulant use, smoking), access-site complications (pseudoaneurysm, retroperitoneal hematoma, arterial occlusion, AV fistula, infection, nerve injury), and procedure-related complications of all DSAs (thromboembolism, iatrogenic dissection, contrast-induced allergy) were systematically recorded.

## Results

**Fig. 2:**
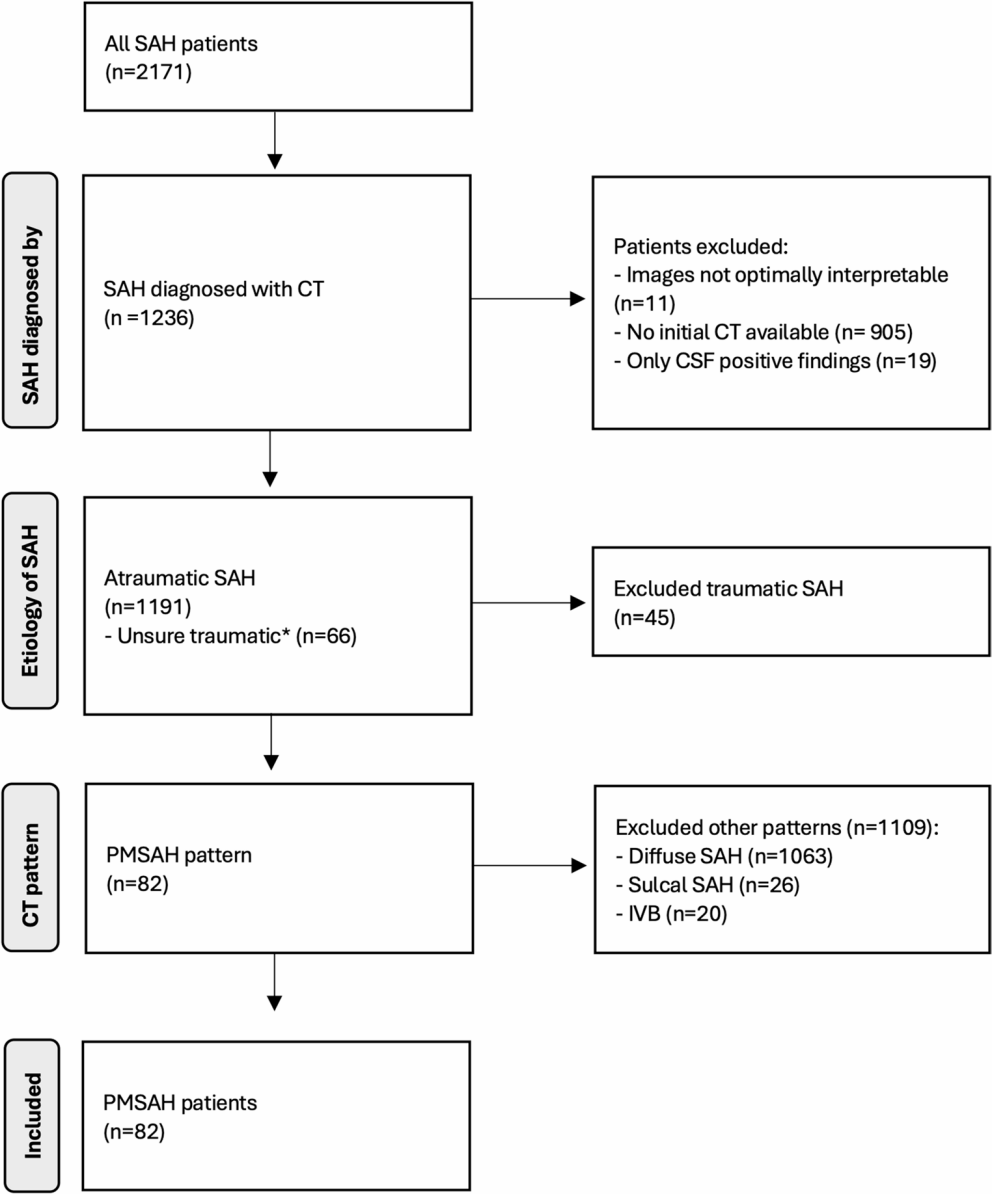
Differentiating perimesencephalic bleeding pattern from all patients with subarachnoid hemorrhage. *CT* computed tomography, *CSF* Cerebrospinal fluid, *IVB* Intraventricular bleeding, *PMSAH* perimesencephalic subarachnoid hemorrhage, *SAH* Subarachnoid hemorrhage *Unsure traumatic: We included patients who had an unobserved fall or were found unconscious where an atraumatic cause for the SAH could not be ruled out

We identified PMSAH patients from a cohort of SAH patients recorded between March 2002 and February 2025 using the algorithm outlined in Fig. 2. Of the 2171 patients, we initially excluded those without a CT-confirmed SAH diagnosis. The non-traumatic SAH cases were then categorized based on bleeding patterns into four groups: (a) PMSAH, (b) diffuse SAH, (c) sulcal SAH, and (d) isolated IVB. Examples of these bleeding patterns are illustrated in Fig. 1.

Table 1 summarizes the distribution of CT patterns among the 1911 patients. Of these, 1063 (89.3%) exhibited a diffuse bleeding pattern, with 288 (27.1%) showing diffuse SAH with intracerebral bleeding. PMSAH was identified in 82 patients (6.9%), sulcal SAH in 26 patients (2.2%), and isolated IVB in 20 patients (1.7%).

**Table 1:** Bleeding patterns

Atraumatic SAH patients (*n* = 1191)	
Diffuse	1063 (89.3%)
Diffuse SAH and ICH	288 (27.1%)
Perimesencephalic	82 (6.9%)
Sulcal	26 (2.2%)
IVB	20 (1.7%)

*ICH* Intracerebral hemorrhage, *IVB* Intraventricular bleeding, *SAH* Subarachnoid hemorrhage

Table 2 summarizes patient baseline characteristics. The median age of the cohort was 52 (IQR 44–57), with 40 out of 82 patients (48.8%) being male. The median hospital stay was 15 days (IQR 11–19), with an in-hospital mortality rate of 1.2% (1/82). The deceased patient developed a brainstem hemorrhage during hospitalization, likely due to hypertension, leading to newly onset left-sided hemiparesis. Consequently, a follow-up DSA could not be performed. The majority of patients (82.9%) presented with a GCS score of 15 or WFNS Grade 1. Other presentations included a GCS score of 14–13 in 5/82 patients (6.1%) and a GCS score below 7 in 1/82 patient (1.2%). Hunt and Hess grading showed: grade 1 in 70.7%, grade 2 in 20.7%, and grade 3 in 1.2% of cases. Radiological evaluation confirmed that all patients had a grade 2 Hemorrhage according to the modified Fisher scale. Arterial hypertension was the most common risk factor for PMSAH, affecting 35/82 patients (42.7%). Additional risk factors included diabetes mellitus (8/82; 9.7%), smoking (7/82; 8.5%), obesity (5/82; 6.1%), antiplatelet therapy (4/82; 4.9%), anticoagulation therapy (3/82; 3.7%), and combined antiplatelet and anticoagulation therapy (1/82; 1.2%). Clinical complications associated with PMSAH included radiologic vasospasm in 10/82 patients (12.2%), spinal drainage in 4/82 (4.9%), hydrocephalus in 3/82 (3.7%), VP shunting in 2/82 (2.4%), and both meningitis and clinical vasospasm in 1/82 patient each (1.2%).

**Table 2:** Baseline characteristics of patients with perimesencephalic subarachnoid hemorrhage

Parameter	Value (*n* = 82)
Demographics	
Age (years), median (IQR)	52 (44–57)
Sex (male)	40 (48.8%)
Clinical data	
Length of stay (days), median (IQR)	15 (11–19)
In-house-mortality	1 (1,2%)
Clinical scores	
GCS	
15	68 (82.9%)
14 − 13	5 (6.1%)
12 − 7	0
< 7	1 (1.2%)
Missing data	8 (9.8%)
Hunt and Hess	
Grade 1	58 (70.7%)
Grade 2	17 (20.7%)
Grade 3	1 (1.2%)
Grade 4	0 (0%)
Grade 5	0 (0%)
Missing data	6 (7.3%)
WFNS	
Grade 1	68 (82.9%)
Grade 2	5 (6.1%)
Grade 3	0 (0%)
Grade 4	0 (0%)
Grade 5	1 (1.2%)
Missing date	8 (9.8%)
Modified Fisher scale	
Grade 0	0 (0%)
Grade 1	0 (0%)
Grade 2	82 (100%)
Grade 3	0 (0%)
Grade 4	0 (0%)
Risk factors	
Arterial Hypertension	35 (42.7%)
Diabetes mellitus	8 (9.7%)
Smoking	7 (8.5%)
Obesity	5 (6.1%)
Coagulation influencing drugs	
Antiplatelet therapy	4 (4.9%)
Anticoagulation	3 (3.7%)
Antiplatelet therapy and anticoagulation	1 (1.2%)
Alcohol	0 (0%)
Drug use	0 (0%)
Clinical events attributable to SAH	
Radiologic Vasospasms	10 (12.2%)
Spinal drainage	4 (4.9%)
Hydrocephalus	3 (3.7%)
VP-Shunt	2 (2.4%)
Clinic Vasospasms	1 (1.2%)
Meningitis	1 (1.2%)
Ventriculostomy	0 (0%)

*GCS* Glasgow coma scale, *IQR* Interquartile range, *SAH* Subarachnoid hemorrhage, *VP* Ventriculoperitoneal, *WFNS* World Federation of Neurosurgical Societies

## Results of diagnostic algorithm

All 82 patients underwent a CT and CTA followed by a DSA, with a median time from initial CT to DSA of less than 24 h (IQR 0–1 days). In most patients (76/82; 92.7%), no bleeding source was identified during the initial DSA. However, an arteriovenous malformation was detected in one patient (1/82; 1.2%). Additionally, five aneurysms (5/82; 6.1%) were identified in the following locations: two in the superior cerebellar artery, one in the posterior communicating artery, one in the basilar artery, and one in the distal internal carotid artery. The arteriovenous malformation and the aneurysms were already identifiable as potential causes of the PMSAH on CTA prior to DSA. Of the 76 PMSAH patients with no identified bleeding source on the initial DSA, a second DSA was performed in 60 cases (60/76; 78.9%) for further investigation of a potential cause. The reasons for not performing additional DSAs in the 16 cases varied. Some patients were transferred and underwent only a single DSA before being relocated back. In other cases, interdisciplinary discussions led to a decision to use MRI or CTA for follow-up instead. In two cases, the patient’s comorbidities, particularly cardiac and pulmonary, were too severe to justify another DSA, with the primary concern being the embolic risk from arrhythmias and the need for intensive medical care due to pneumonia. The median interval between the first and second DSA for the 60 patients was 14 days (IQR 12–14). In most cases, no bleeding source was detected (58/60; 96.7%). However, an aneurysm was identified in 2 out of 60 patients (3.3%):

### Case 1

**Fig. 3:**
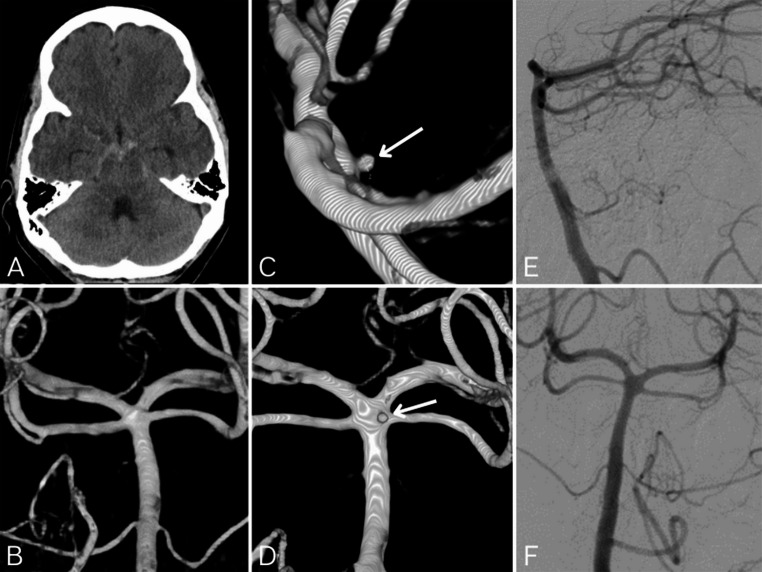
A. Non-contrast cranial CT showing evidence of a perimesencephalic subarachnoid hemorrhage **B.** First digital subtraction angiography (DSA) with 3D rotational angiography (frontal view) from the left vertebral artery, revealing no bleeding source such as an aneurysm **C**,** D.** Second DSA performed 10 days later with 3D rotational angiography from the left vertebral artery: (C = lateral view; D = frontal view). A small dorsally oriented aneurysm (0.8 × 1 mm) of the distal basilar artery is identified (arrows) **E**,** F.** Second DSA with standard angiographic series from the left vertebral artery: (E = lateral projection; F = frontal projection). Despite 3D rotational angiography, the small basilar artery aneurysm remains undetectable on standard projections

A 45-year-old patient presented with a sudden onset of occipital Headache while playing tennis. There were no signs of meningeal irritation or other neurological deficits. The GCS score was 15, Hunt and Hess grade 1, and the modified Fisher score was 2. Non-contrast CT revealed a PMSAH. Within 24 h, a complete panangiography was performed, including catheterization of the bilateral internal carotid arteries, external carotid arteries, and vertebral arteries. Additionally, a 3D rotational angiography (3DRA) was conducted from the dominant vertebral artery and both internal carotid arteries. No source of bleeding was identified (Fig. 3). A follow-up MRI, performed four days later, also showed no evidence of a bleeding source. Ten days later, a second DSA revealed a small saccular aneurysm (0.8 × 1 mm) located at the dorsal basilar artery, at the origin of the right superior cerebellar artery. The aneurysm was only visible on 3D rotational angiography and could not be identified on standard angiographic series from the vertebral artery. After interdisciplinary discussion and thorough risk-benefit analysis, the decision was made to forgo endovascular treatment. The patient was discharged without neurological deficits after a 20-day hospital stay. Two follow-up MRIs showed no abnormalities.

### Case 2

**Fig. 4:**
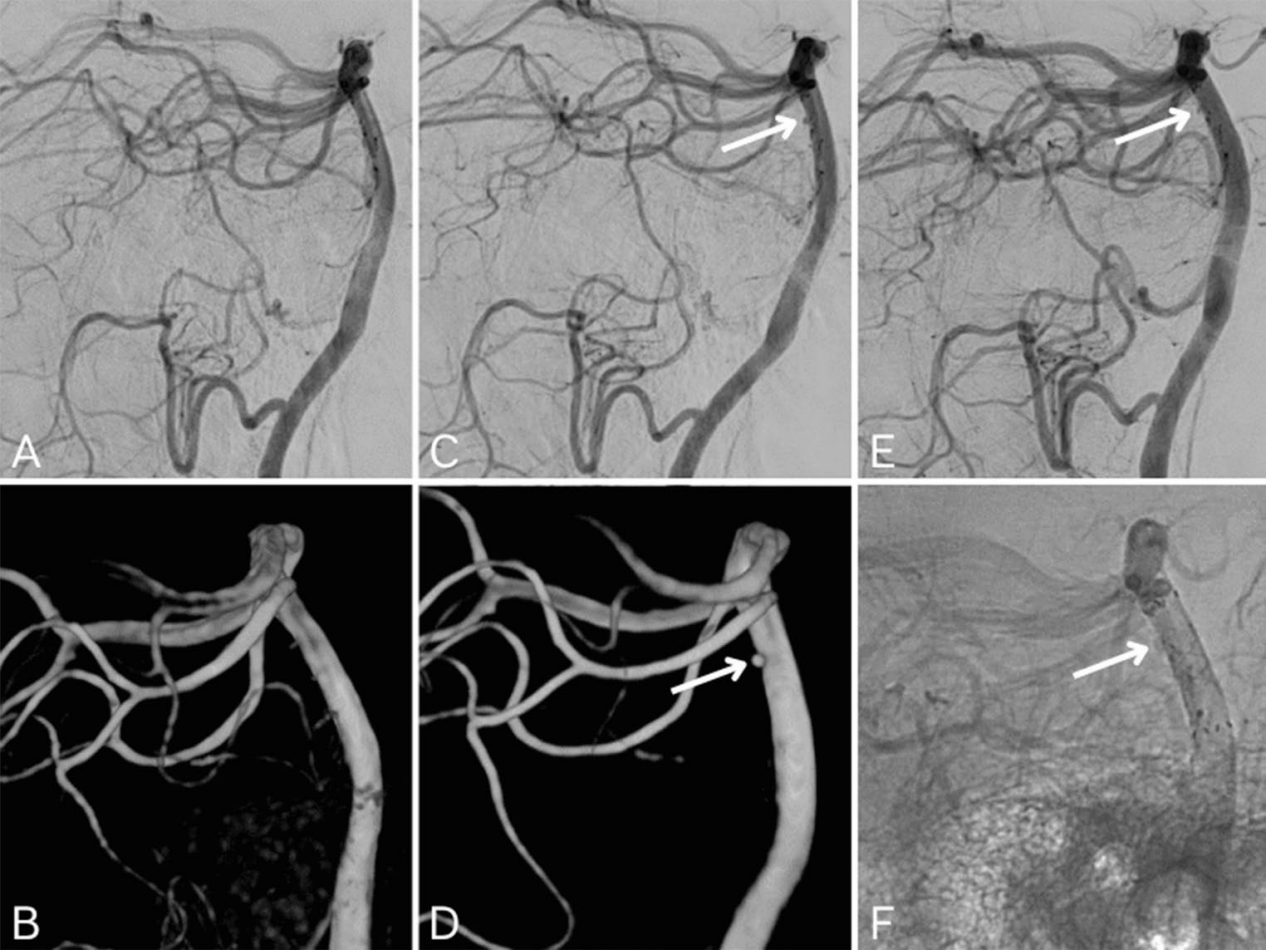
**A**,** B.** First digital subtraction angiography (DSA) with standard series (**A**) and 3D rotational angiography (**B**) from the left vertebral artery, showing no evidence of an aneurysm **C**,** D.** Second DSA performed 14 days later, revealing a dorsally oriented aneurysm of the basilar artery (arrows), measuring 1 × 1.3 mm. (C = lateral projection of the standard series; D = lateral projection of the 3D rotational angiography) **E**,** F.** DSA following flow diverter implantation (FRED X 3.5 × 13 mm, MicroVention, Aliso Viejo, California, USA) in the basilar artery, bridging the aneurysm and demonstrating low residual perfusion immediately postintervention (arrows; E = lateral projection of the standard series; F = corresponding bone image for enhanced visualization of the flow diverter)

A 53-year-old patient presented with a sudden onset of Headache upon waking, accompanied by neck stiffness and signs of meningism. No neurological deficits were observed. Upon admission, the GCS score was 15, Hunt and Hess grade 1, and the modified Fisher score was 2. Non-contrast CT revealed a PMSAH. Initial panangiography, including imaging of the bilateral internal carotid arteries, external carotid arteries, vertebral arteries, and 3D rotational angiography showed no source of bleeding (Fig. 4). Given the patient’s use of a non-vitamin K oral anticoagulant, a hypertensive origin for the Hemorrhage was initially suspected. An MRI performed within five days of the DSA revealed no bleeding source. The second DSA, conducted 14 days later, identified a small dorsally directed aneurysm (1 × 1.3 mm) of the basilar artery. This aneurysm was visible on both 3D rotational angiography from the left vertebral artery and the standard angiographic series. Following medical preparation with acetylsalicylic acid and ticagrelor, interventional treatment was performed three days later with the implantation of a FRED X 3.5 × 13 mm flow diverter (MicroVention, Aliso Viejo, California, USA). The device fully covered the aneurysm, with minimal residual perfusion remaining after the intervention. Two follow-up DSAs over the subsequent weeks showed gradual occlusion of the aneurysm, and after five weeks, the aneurysm was classified as fully excluded (O’Kelly-Marotta grading scale, grade D). The patient was discharged without neurological deficits after a 34-day hospital stay.

In 5 out of 58 patients (8.6%) where no source of bleeding was identified in the first two DSAs, a third DSA was performed. The median interval between the second and third DSA in these patients was 31 days (IQR 12–185.5). In one of the five cases, the third DSA was conducted after 308 days due to a suspected basilar artery fenestration, which was not confirmed. No bleeding source was detected in any of these five cases.

## Complications of DSA

A total of 147 DSAs were performed on 82 patients to identify a bleeding source in PMSAH, excluding procedures where DSA was not intended for this purpose. Of these, three procedure-related complications occurred (2%). In the first case, the patient experienced pain at the puncture site, cribriform paraesthesia in the foot, and exertional pain. Although a thigh hematoma was initially suspected, MRI findings did not confirm this diagnosis. The symptoms were likely attributable to the use of an arterial closure system (6 F Angio-Seal™, St Jude Medical, St Paul, Minnesota, USA). In another patient, supratentorial microembolisms were observed on MRI following the first DSA. These findings were likely aggravated by the patient’s preexisting high-grade internal carotid artery stenosis, leading to the decision not to perform further DSAs. The third complication involved a well-demarcated cerebellar infarction in the territories of the anterior inferior and posterior inferior cerebellar arteries, detected on MRI after the first DSA. This infarction was attributed to the DSA, likely caused by elongated, arteriosclerotically altered vessels with no evidence of vasospasm. Despite this, a second DSA was conducted for this patient.

## Discussion

Our findings reaffirm the benign nature of PMSAH [8–10]. Most patients presented in good neurological condition (GCS 15 in 82.9%), and the in-hospital mortality rate was low (1.2%). Relevant complications such as hydrocephalus (3.7%) and clinically significant vasospasm (1.2%) were rare.

In our cohort, 147 DSAs were performed, including 60 follow-up studies. In six cases, a therapeutically relevant aneurysm, identified as the source of bleeding in PMSAH, was detected in the first DSA and was already visible in the initial CTA. This underscores the adequacy of CTA for identifying aneurysms in these cases. In the repeat DSA, two previously undetected aneurysms (3.3%) were identified. However, three DSA-related complications (2%) occurred, including cerebellar infarction, supratentorial microemboli, and a local puncture complication.

These findings are consistent with those from more extensive studies. One meta-analysis study reported that aneurysms were identified in only 0.8% of follow-up DSAs among 1,031 patients (upon careful review, the accuracy of some follow-up-reported aneurysms was called into question), and another study found a pooled detection rate of aneurysm of 0.3% [11, 15]. Several additional studies observed no new aneurysms with repeat DSA [12–14]. In patients with PMSAH, the complication rate associated with DSA ranges from 1.4 to 1.6% [16, 17]. These results highlight that repeat DSA in PMSAH often yields a low detection rate for aneurysms related to the bleeding source while also underscoring the associated complication risks of DSAs.

Our institution’s diagnostic algorithm for patients with PMSAH involves an initial CTA followed by DSA and often a repeated DSA after 10–14 days. In our study, both aneurysms were not visible on initial CTA, DSA, or 3DRA but were identified on repeat 3DRA several days later. Contributing factors include their small size (0.8 × 1 mm and 1 × 1.3 mm, respectively), their location in the posterior circulation (basilar artery) near the skull base, and the fact that the aneurysms were already fully thrombosed at the time of the initial 3DRA. These factors raise important considerations regarding the limitations of imaging techniques in SAH, particularly in the context of PMSAH.

In cases of PMSAH, it may be reasonable to consider dual-source CTA (DE-CTA), Ultra-High-Resolution CTA (UHR-CTA), or UHR photon-counting CTA (UHR PCT-CTA) as the primary diagnostic tool instead of standard CTA. These advanced CTA technologies provide significantly improved spatial resolution and reduce beam-hardening artifacts, which enhances visualization in challenging areas such as the skull base [18–20].

This is particularly important for detecting small aneurysms under 3 mm in size. While standard CTA shows a sensitivity of approximately 61% for aneurysms ≤ 3 mm and around 96% for larger aneurysms [21], DE-CTA has demonstrated substantially higher sensitivity: 80%−91.7% for aneurysms ≤ 3 mm, and 93.8%−100% for aneurysms > 3 mm, respectively [22, 23].

UHR-CTA significantly enhances diagnostic accuracy in detecting intracranial aneurysms in patients with aneurysmal subarachnoid Hemorrhage, reaching 100% patient-level and 96–98% segment-level sensitivity versus 82–88% and 76–82% for Normal-Resolution CTA, with added benefits of superior image quality and reduced radiation exposure [24].

In participants with suspected cerebrovascular disease and unruptured aneurysms, UHR photon-counting CTA (UHR PCT-CTA) demonstrated significantly higher sensitivity (98.0% vs. 72.0%), specificity (96.7% vs. 86.7%), and overall accuracy (97.3% vs. 80.0%) for aneurysm detection compared with Standard-Resolution photon-counting CTA (SR PCD-CTA). Beyond improved detection, UHR PCT-CTA also enabled superior morphological characterization, including the identification of aneurysm irregularities, wall calcifications, and intra-aneurysmal hypointensity suggestive of thrombus [25].

To date, no direct comparison between UHR-CTA and conventional CTA regarding the detection rate of small aneurysms has been reported.

Given these techniques’ non-invasive nature and increasing availability, their use can improve early diagnostic accuracy and potentially reduce the need for invasive procedures.

If a DSA is deemed necessary, it should include a 3DRA of the vertebral arteries. 3DRA increases sensitivity by enabling detailed assessment of vascular anatomy from multiple angles. It provides important information about aneurysm morphology, such as neck configuration, shape, and relationship to adjacent vessels, which is critical for determining treatment strategies [26]. Nevertheless, even DSA may miss aneurysms due to vasospasm, which reduces contrast filling in the acute phase, or thrombosis, which can obscure aneurysm visibility and lead to false-negative findings [27–29].

Vessel wall magnetic resonance imaging (VW-MRI) is gaining increasing attention for its potential to detect wall changes in thrombosed aneurysms and may be particularly valuable in the assessment of angiogram-negative SAH cases [30, 31]. Initial studies using VW-MRI in NPMSAH showed abnormalities in 63.6% of negative angiographic cases, though without therapeutic consequences [32]. Studies further support a venous origin, with PMSAH patients more often exhibiting primitive venous drainage patterns and side-specific correlation to the hemorrhage [33]. This suggests that advanced arterial imaging, like VW-MRI, may offer limited benefit in typical PMSAH cases, in contrast to other angiogram-negative SAH types where hidden arterial lesions remain a concern.

Given this, VW-MRI may best be used after an initial high-quality CTA (ideally dual-source or photon-counting) and before proceeding to DSA. If VW-MRI shows no suspicious findings, the necessity of an invasive DSA should be reevaluated in an interdisciplinary setting. In such cases, a stepwise diagnostic approach may help avoid unnecessary invasive procedures while maintaining diagnostic safety.

A key clinical question remains: Does identifying a bleeding source in PMSAH always necessitate intervention?

In our study, repeat DSA identified two basilar artery perforator aneurysms (BAPAs). One aneurysm was treated with flow diversion (case 2), while the other was managed conservatively (case 1). Both patients experienced favorable clinical outcomes, regardless of the chosen therapeutic approach.

BAPAs are exceedingly rare and frequently overlooked due to their small size (85% measure 3 mm or less), tendency toward thrombosis, and originating from fragile perforating arteries. Their detection often requires 3DRA or vessel wall imaging, with wall enhancement as an important diagnostic marker [34, 35]. Despite advanced imaging techniques, studies report that BAPAs are identified on the initial DSA in only 28% and 50% [35, 36]. Due to their rarity and complex anatomy, no clear treatment guidelines exist. Both endovascular approaches, including coiling and flow diversion, and conservative management strategies, are being considered [35].

Flow diversion has proven effective in achieving angiographic occlusion; however, it carries significant risks, including ischemic complications and the need for dual antiplatelet therapy. In one multicenter study, ischemic events have been reported in up to 28% of cases, documenting a long-term mortality rate of 13% [35]. Another study reported additional complications, including in-stent thrombosis in 40% of patients, perforator strokes in 20%, and the need for surgical intervention related to external ventricular drainage in 20% [37]. In contrast, patients with ruptured BAPAs who were managed conservatively experienced a rebleeding rate of 11.1%. Spontaneous occlusion was observed in 55.5% of these cases. However, 27.8% of patients developed spontaneous perforator strokes, with outcomes ranging from mild to severe [37]. Based on these findings, one study concluded that initial conservative management may be appropriate, especially in clinically stable patients, with endovascular treatment reserved for cases of rebleeding, aneurysm enlargement, or failure to demonstrate spontaneous occlusion [37].

Without randomized trials or more extensive case-control studies, the optimal therapeutic approach for BAPAs remains uncertain and will likely be shaped by future evidence.

A comparison of our cases highlights the uncertainty surrounding the optimal management of these aneurysms, as repeated DSA did not lead to definitive therapeutic decisions, and the clinical course may well have been similar even without their detection or intervention.

Considering that all relevant sources of hemorrhage were already identified by CTA in our six cases, and that BAPAs may not entail any therapeutic consequences, it appears that DSA may not be necessary in the diagnostic evaluation of PMSAH.

This study is limited by its retrospective, single-center design and lack of a control group, restricting the ability to generalize the findings. The small number of patients with PMSAH also limits statistical comparisons and may introduce selection bias. Additionally, in six cases the Hunt and Hess scale, and in eight cases GCS, were not documented. The lack of initial medical reports prevented documentation of the clinical presentation at admission, which may be attributed to inconsistent documentation practices in cases dating back to 2002.

## Conclusion

Considering our findings, all relevant sources of hemorrhage were identified by the initial CTA. Follow-up DSA identified two BAPAs, leading to a flow diverter placement in one case (Case 2). Consistent with our observations, no clear therapeutic benefit over conservative management (Case 1) was evident, and no standardized treatment protocol for BAPAs currently exists. Given the low diagnostic yield and procedural risks, combined with the benign course of PMSAH and the availability of advanced non-invasive imaging (DE-CTA, UHR-CTA, UHR PCT-CTA, vessel wall MRI), repeat DSA should not be considered standard practice in PMSAH. Instead, its use should be reserved for selected cases with atypical clinical or radiological features, based on an individualized risk-benefit assessment and guided by interdisciplinary decision-making. Further research and advances in imaging techniques will be essential to determine the role of DSA in PMSAH, particularly in identifying rare entities like BAPA, where therapeutic strategies remain unclear and formal guidelines are not yet established.

## Data Availability

The data that support the findings of this study are not publicly available due to institutional and ethical restrictions, but may be available from the corresponding author on reasonable request and with permission of the University Hospital Essen.
